# Comparative Genomic Analysis of 45 Type Strains of the Genus *Bifidobacterium*: A Snapshot of Its Genetic Diversity and Evolution

**DOI:** 10.1371/journal.pone.0117912

**Published:** 2015-02-06

**Authors:** Zhihong Sun, Wenyi Zhang, Chenyi Guo, Xianwei Yang, Wenjun Liu, Yarong Wu, Yuqin Song, Lai Yu Kwok, Yujun Cui, Bilige Menghe, Ruifu Yang, Liangping Hu, Heping Zhang

**Affiliations:** 1 Key Laboratory of Dairy Biotechnology and Engineering, Education Ministry of China, Inner Mongolia Agricultural University, Hohhot, Inner Mongolia, China; 2 State Key Laboratory of Pathogen and Biosecurity, Beijing Institute of Microbiology and Epidemiology, Beijing, China; 3 Synergetic Innovation Center of Food Safety and Nutrition, Northeast Agricultural University, Harbin, 150030, China; 4 Consulting Centre of Biomedical Statistics, Beijing, 100850, China; University of Ulm, GERMANY

## Abstract

Bifidobacteria are well known for their human health-promoting effects and are therefore widely applied in the food industry. Members of the *Bifidobacterium* genus were first identified from the human gastrointestinal tract and were then found to be widely distributed across various ecological niches. Although the genetic diversity of *Bifidobacterium* has been determined based on several marker genes or a few genomes, the global diversity and evolution scenario for the entire genus remain unresolved. The present study comparatively analyzed the genomes of 45 type strains. We built a robust genealogy for *Bifidobacterium* based on 402 core genes and defined its root according to the phylogeny of the tree of bacteria. Our results support that all human isolates are of younger lineages, and although species isolated from bees dominate the more ancient lineages, the bee was not necessarily the original host for bifidobacteria. Moreover, the species isolated from different hosts are enriched with specific gene sets, suggesting host-specific adaptation. Notably, bee-specific genes are strongly associated with respiratory metabolism and are potential in helping those bacteria adapt to the oxygen-rich gut environment in bees. This study provides a snapshot of the genetic diversity and evolution of *Bifidobacterium*, paving the way for future studies on the taxonomy and functional genomics of the genus.

## Introduction

*Bifidobacterium* is a genus of Gram-positive, non-spore-forming bacteria that are commonly found in the gastrointestinal tract of humans and animals [1, 2]. In 1900, the first *Bifidobacterium* strain was isolated by Tissier from the feces of a breast-fed infant and was named *Bacillus bifidus* [3]. It was not until 1924 that a Danish microbiologist named Orla-Jensen classified it as a separate species within the genus *Bifidobacterium* [4]. Currently, this genus contains 48 recognized taxa (http://www.bacterio.net/bifidobacterium.html), which are divided into six main phylogenetic groups, namely, *B*. *adolescentis*, *B*. *longum*, *B*. *pullorum*, *B*. *asteroides*, *B*. *pseudolongum* and *B*. *boum* [5]. Possessing a fermentative phenotype of metabolism, bifidobacteria are able to produce acid from a variety of carbohydrates via the fructose-6-phosphate phosphoketolase pathway.

Bifidobacteria are often associated with health-promoting effects, either as an endogenous member of the intestinal microbiota (e.g., protection and immunomodulation) or as allochthonous probiotics species (e.g., diarrhea prevention), which has led to their wide application in food and probiotic products [6–11]. Furthermore, there is growing interest in exploring the diversity of the bifidobacterial population within the human gut microbiota [12], as it has been revealed by both culture-dependent and pyrosequencing analyses that *Bifidobacterium* is the predominant genus in the infant gut [13, 14]. Moreover, multiple bifidobacterial species co-occur in the same environment [14]. Thus, bifidobacteria may exert strong influence in not only colonic health but also in the establishment of the gut environment and shaping of other microbiota successors. Further information underlying the interactions between *Bifidobacterium* and the human gut was reported by Avershina et al. [15], who found a highly structured and age-related succession of bifidobacterial species within a large, unselected healthy cohort of mothers and infants. Among the key bifidobacterial taxa (*B*. *adolescentis*, *B*. *bifidum*, *B*. *dentium*, *B*. *breve*, and *B*. *longum*) identified in the human gut, *B*. *longum* was found to be one of the most central bifidobacteria [15]. Interestingly, in children who harbored gut *B*. *longum* subsp. *infantis* at 4 months of age, *B*. *longum* subsp. *longum* was also detected later in life [15]. It has been revealed by comparative genomic studies that the former subspecies is specialized in utilizing human milk oligosaccharides (HMOs), whereas the latter preferentially metabolizes plant-derived carbon [16]. Such a phenomenon may suggest the role of HMOs in modulating the gut microbial composition at specific life stages. An intriguing study from Turroni et al. [17] reported some novel *Bifidobacterium* taxa after assessing the complexity and diversity of the human mucosa-adherent bifidobacterial population by molecular methods. Thus, it is likely that more extensive genomic analyses will allow a deeper understanding of bifidobacterial diversity, and such studies will reveal host-bifidobacterial interactions in a more precise manner.

Conventionally, the taxonomy of *Bifidobacterium* has been based on biochemical tests and has progressed rapidly due to the development of modern microbial population genetics, ecology and genomics [18]. At present, genome-based approaches and methods dependent on defined marker genes including 16S rRNA and multiple housekeeping genes (e.g., *recA*, *tufA*, *gro*EL, *clp*C, *fusA*) have been used for detecting and identifying bifidobacterial species [19–22]. Nonetheless, the resolution of bacterial classification that merely relies on a single or few genetic marker(s) is relatively low [18]. Indeed, phylogenetic trees built using a set of orthologous genes shared by the previously published nine complete bifidobacterial genomes have provided more robust data on the relationships between different species [23]. Similarly, Ventura et al [24] clearly confirmed a higher discriminatory power of a phylogenetic tree constructed using a genome-wide multilocus approach that analyzed the pairwise distances and standard deviation of orthologous genes of different bifidobacterial strains in comparison to that built with the 16S rRNA gene. However, due to the sampling bias toward *Bifidobacterium* species of biotechnological importance, the number of strains that have been sequenced thus far is still rather limited, and a large gap remains to be filled with regard to their phylogeny [25].

In the present study, a total of 45 type strains, covering 93.75% of the genus *Bifidobacterium*, were comparatively analyzed. Our aim was to reassess the genetic diversity of the genus and to infer the phylogenetic relationship among its members. Our results revealed interesting clues regarding the niche jumping of *Bifidobacterium* species, which most likely originated from the insect host environment before spreading to humans.

## Materials and Methods

### Bacterial strains and DNA extraction

The genome sequences of 45 type strains of bifidobacterial species (subspecies) defined by LPSN (http://www.bacterio.net/) were analyzed. Among them, 41 genomes were deciphered in this research, and 4 genomes published previously were acquired from GenBank (*B*. *animalis* subsp. *animalis* ATCC 25527, *B*. *animalis* subsp. *lactis* ATCC 27653, *B*. *adolescentis* ATCC 15703 and *B*. *longum* subsp. *longum* JCM 1217). The type strain of *B*. *dentium* (known as BD1, ATCC27534) was sequenced both in previous [26] and the present work (identification number: DSM 20436), and its de novo sequenced genome in this research was used in further analyses. The 45 type strains covered 38 *Bifidobacterium* species and 7 subspecies, including 1 subspecies of *B*. *animalis* and 2 subspecies each of *B*. *longum*, *B*. *pseudolongum*, and *B*. *thermacidophilum* (S1 Table and S2 Table).

Among the 41 strains sequenced in this research, 40 were obtained from the German Collection of Microorganisms and Cell Cultures (DSMZ), and one strain was obtained from the Japan Collection of Microorganisms (JCM). DNA extraction was performed using a bacterial DNA extraction kit (OMEGA D3350–02) according to the manufacturer’s instructions. Briefly, after overnight incubation of the type strains under anaerobic conditions (80% N_2_, 10% H_2_ and 10% CO_2_) in MRS broth at 37 ℃, the cells were collected by centrifugation at 12,000 × g for 30 seconds and subjected to lysozyme cycles for cell lysis. Next, 0.25 M EDTA was added and mixed well, and the lysate was incubated on ice for 5 min. This was followed by the addition of a series of buffers provided in the kit. To elute the DNA, 100–200 μL of deionized water was added. The integrity of DNA was checked by electrophoresis on 1% agarose gels. Quality control was subsequently carried out on the purified DNA samples. Genomic DNA was quantified using a TBS-380 fluorometer (Turner BioSystems Inc., Sunnyvale, CA). High-quality DNA samples (OD260/280 = ~1.8–2.0, >6 μg) were utilized to construct a fragment library (200 to 300 bp).

### Sequencing, assembly, prediction and annotation of coding sequences (CDSs)

Whole-genome sequencing was performed using an Illumina HiSeq 2000 by generating paired-end libraries with insert sizes of 300 bp following the manufacturer’s instructions. For the Illumina pair-end sequencing of each strain, at least 3 μg genomic DNA was used for sequencing library construction. Paired-end libraries with insert size of ~300 bp were prepared following Illumina’s standard genomic DNA library preparation procedure. Purified genomic DNA was sheared into smaller fragments to a desirable size using Covaris fragmentation, and blunt ends were generated using T4 DNA polymerase. After adding an ‘A’ base to the 3′ end of the blunt phosphorylated DNA fragments, adapters were ligated to the ends. The desired fragments were purified by agarose gel electrophoresis before being selectively enriched and amplified by PCR. An index tag was introduced via an adapter during PCR. A library quality test was then performed, and the qualified Illumina pair-end library was used for Illumina Hiseq 2000 sequencing. The read lengths were 100 bp, and an average of 788 Mb of high-quality data were generated for each strain, corresponding to a sequencing depth of 212- to 491-fold (S2 Table).

The pair-end reads were first *de novo* assembled using SOAPdenovo v2 [27]; local inner gaps were then filled, and single base errors were corrected using the software GapCloser (http://sourceforge.net/projects/soapdenovo2/files/GapCloser/). A uniform annotation pipeline was applied for analyzing both the newly sequenced genomes herein and four publicly available genomes. CDSs were predicted using Prodigal [28, 29], and the functional annotation of predicted genes was performed by alignment against a non-redundant public database and the cluster of orthologous groups (COG) database of NCBI using amino acid sequences. The individual genome assemblies of the 41 strains have been deposited in National Center for Biotechnology Information. The accession numbers are listed in S2 Table.

### Construction of core- and pan-gene families

Similar to the situation for a genus, a large genetic distance between species will result in a relatively great variation in the sequences of homologous genes. Therefore, we used the concept of “gene family" to replace the generally used “gene” to explore the genome content of the genus *Bifidobacterium*. The gene family of bifidobacteria was defined based on a previous report [30]. Briefly, a pair of genes was assigned to the same gene family when the identity value of their amino acid sequences was above 50% and more than 50% of the amino acid sequence of the longer gene was homologous to the other one. The gene family prediction was validated by a tblastn [31] search to avoid members of a gene family not being identified in a genome when the corresponding ORF was not predicted or the 5’ was not properly predicted, leading to ORFs with an incorrect length. For the construction of the pan-gene family, we first grouped all of the predicted genes into a possible gene family for each genome and then accumulated the gene families of all genomes to obtain their union set. The core-gene family was constructed by counting the number of commonly shared gene families within all of the genomes, and 402 core-gene families were identified. The sequence of the longest gene from each core-gene family was then selected as the representative sequence for functional annotation and phylogenetic reconstruction (S3 Table).

### Calculation of the genome-average nucleotide identity (ANI) and average amino acid identity (AAI)

The pair-wise ANI values across the newly sequenced bifidobacterial strains were calculated according to the method proposed by Goris et al. [32]. Using the predicted protein sequence for each genome, we calculated pairwise AAIs based on blastp. The items in the blastp results that (i) showed less than 30% identity, (ii) an alignment length less than 30 aa, or (iii) less than 70% of the entire length of the protein were removed. When one gene matched with multiple sequences, only the best hit was selected to calculate the average value of the identity.

### Phylogenetic analysis

To build a complete bacterial phylogenetic tree, we selected 426 representative genomes of different genera spanning 27 phyla, as provided by the software AMPHORA2 [33]. When more than one genome was included in one genus in AMPHORA2, we randomly selected one genome for further analysis. Furthermore, AMPHORA2 provided 31 commonly shared marker genes (S3 Table) across the 426 selected representative strains (S4 Table) and the 45 bifidobacterial genomes analyzed herein, and we used these 31 marker genes to construct the phylogenetic tree of the 45 *Bifidobacterium* strains and 426 representative genomes (S1 Fig.).

We employed the amino acid sequences of 402 core genes of the genus *Bifidobacterium* (covering the 31 marker genes, S3 Table) to construct the phylogenetic tree of the 45 *Bifidobacterium* species. We aligned the amino acid sequences of these genes using MUSCLE v3.8.31 [34] and then constructed a maximum likelihood tree based on the concatenated alignments using the software PHYML with WAG model and 500 bootstrap iterations [35]. The most recent common ancestor (MRCA) of the bifidobacterial tree was defined according to its corresponding position in the tree of bacteria. The maximum likelihood tree of bifidobacteria, *Gardnerella* and *Brachybacterium* was built using the same protocol based on 185 common genes shared by 47 genomes of these three genera.

The number of nodes to the MRCA was counted as the number of nodes on the shortest path from a strain (tip of the tree) to the MRCA. A Wilcoxon Two-Sample test was performed to compare the node numbers between isolates from human and bee.

## Results and Discussion

### General genomic characteristics of the *Bifidobacterium* genus

**Fig 1:**
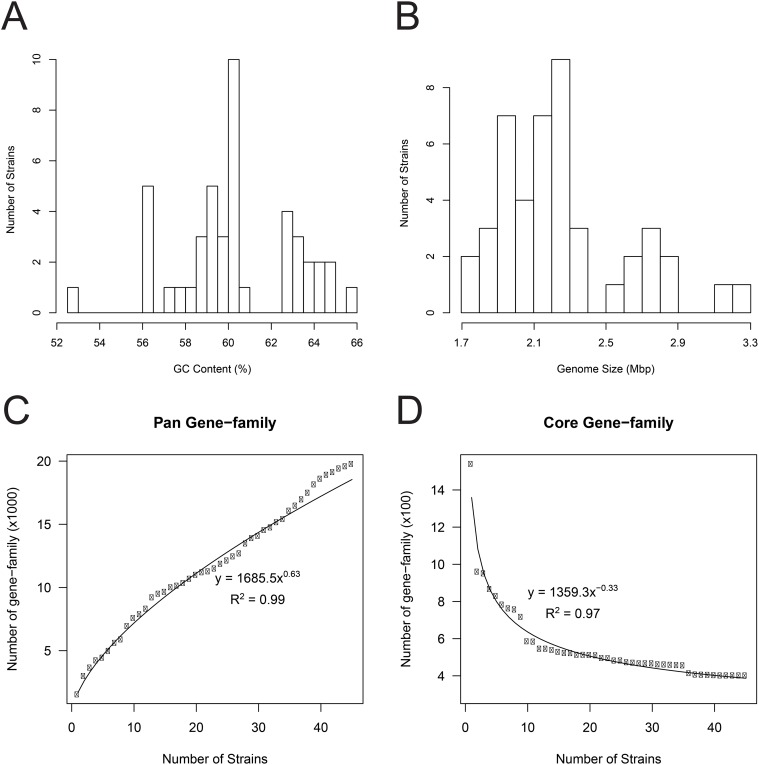
Genomic characteristics of the genus *Bifidobacterium*. A and B. The frequency distribution of the GC content (A) and genome size (B) across genomes of 45 type strains. The y-axis represents the number of strains. C and D. Size of pan- and core-gene families of *Bifidobacterium* versus the number of genomes. The size of pan- and core-gene families (dots) followed the power function (curves) with an increasing number of genomes using 45 bifidobacteria strains.

The genus *Bifidobacterium* exhibits a high G+C content that ranges from 52.8% to 65.8% (Fig. 1A and S2 Table). The genome sizes of *Bifidobacterium* were found to vary significantly [36], ranging from 1.7 Mb (*B*. *indicum*, DSM 20214) to over 3.3 Mb (*B*. *biavatii*, DSM 23969) (Fig. 1B), with 1,369 to 2,564 predicated coding genes, respectively (S2 Table). The pan-genome in this work contained more than 20,000 gene families and grew continuously, with an average of 379 more gene families with the addition of each genome (Fig. 1C), indicating an open pan-genome of the genus *Bifidobacterium*.

**Fig 2:**
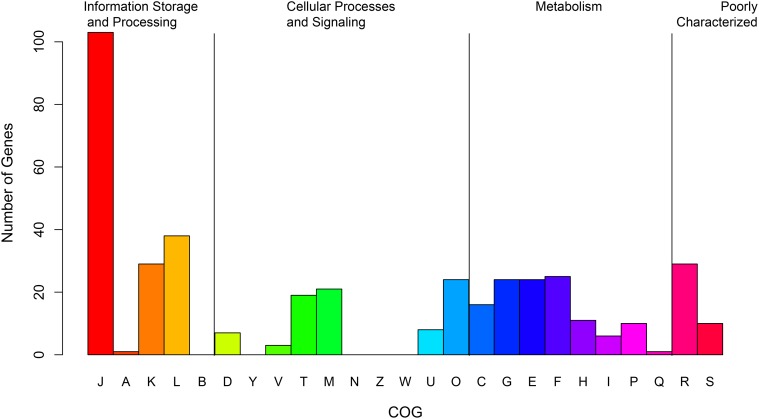
Distribution of functional categories of core gene families in the genus *Bifidobacterium*. COG Functional Classification Description—Information Storage and Processing: [J] Translation, ribosomal structure and biogenesis; [A] RNA processing and modification; [K] Transcription; [L] Replication, recombination and repair; [B] Chromatin structure and dynamics. Cellular Processes and Signaling: [D] Cell cycle control, cell division, chromosome partitioning; [Y] Nuclear structure; [V] Defense mechanisms; [T] Signal transduction mechanisms; [M] Cell wall/membrane/envelope biogenesis; [N] Cell motility; [Z] Cytoskeleton; [W] Extracellular structures; [U] Intracellular trafficking, secretion, and vesicular transport; [O] Posttranslational modification, protein turnover, chaperones. Metabolism: [C] Energy production and conversion; [G] Carbohydrate transport and metabolism; [E] Amino acid transport and metabolism; [F] Nucleotide transport and metabolism; [H] Coenzyme transport and metabolism; [I] Lipid transport and metabolism; [P] Inorganic ion transport and metabolism; [Q] Secondary metabolites biosynthesis, transport and catabolism. Poorly Characterized: [R] General function prediction only; [S] Function unknown.

In contrast to the pan-gene families, the number of core-gene families shared across different species decreased sharply as the number of genomes increased, reaching a minimum value of 402 for all 45 genomes analyzed (Fig. 1D). The majority of these core genes appeared to encode proteins that are involved in basic cell maintenance (S3 Table for the list of core genes); the functional categories of these core genes were attributed according to the Cluster of Orthologous Group (COG) classification. As indicated in Fig. 2, 25.18% of the core genes belong to the category of Translation, ribosomal structure and biogenesis ([J]), followed by the categories of Replication, recombination and repair ([L]) (9.29%) and Transcription ([K]) (7.09%). Accordingly, each other functional category only included a very limited number of core genes.

### Defining the root of the genus *Bifidobacterium*

To examine the phylogenetic position of the genus *Bifidobacterium* within the domain Bacteria, we constructed a tree of bacteria using 45 bifidobacterial genomes and 426 other selected genomes representing 426 genera from 27 phyla of bacteria (S1 Fig.). Furthermore, we built a maximum likelihood tree (MLtree) using 45 bifidobacteria together with the representative genomes of two closely related genera, *Gardnerella* and *Brachybacterium*, which served as the outgroup control (S2 Fig.).

Both analyses supported that all bifidobacterial species were descended from the same most recent common ancestor (MRCA), forming a distinct lineage in the phylum *Actinobacteria*. The highly robust MLtrees indicated that the lineage including the *B*. *asteroides* group is closest to the outgroup strains and thus should be the most ancient lineage of bifidobacteria. This was in contrast to the previous observation by 16S rRNA sequence analysis that *B*. *subtile* was the deepest branch of bifidobacteria [24]. Such a discrepancy might be caused by homologous recombination occurring at the 16S rRNA locus, which obscured the vertical genetic signals and led to a biased observation.

### Evolution of *Bifidobacterium*

**Fig 3:**
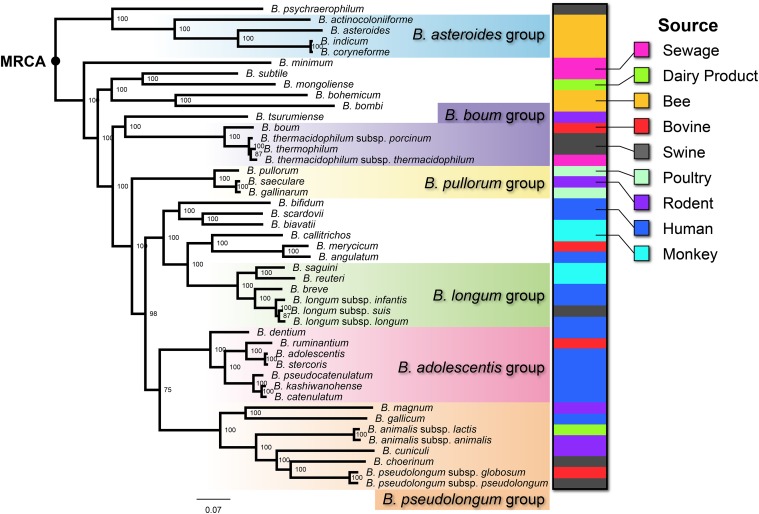
Maximum likelihood tree of 45 *Bifidobacterium* species based on 402 core gene families. The tree was built using PHYML with 500 bootstrap iterations. The scale bar represents substitutions per SNP site, and the numbers near nodes are the percentages of supported bootstrap values. The shading color indicates previously defined phylogroups [24]. The color chart at the right side of the tree indicates the isolated source of the corresponding strains.

To infer phylogenetic relationships across species within the genus *Bifidobacterium*, we constructed an MLtree of 45 type strains based on the concatenated amino acid sequences of 402 core genes (Fig. 3). The tree is robust, as most of the nodes were supported by 100% of the bootstrap iterations, with a slightly lower supported proportion (75%–98%) observed only in four nodes.

According to the MRCA position, we were able to order speciation events during the genealogy of bifidobacteria. Our data showed that the *B*. *asteroides* group belongs to the most ancient lineage of bifidobacteria, as mentioned above, and that the *B*. *boum* group and the *B*. *pullorum* group emerged after an interval with several single species. The three remaining phylogroups, *B*. *longum* group, *B*. *adolescentis* group and *B*. *pseudolongum* group, clustered within the younger lineages.

**Fig 4:**
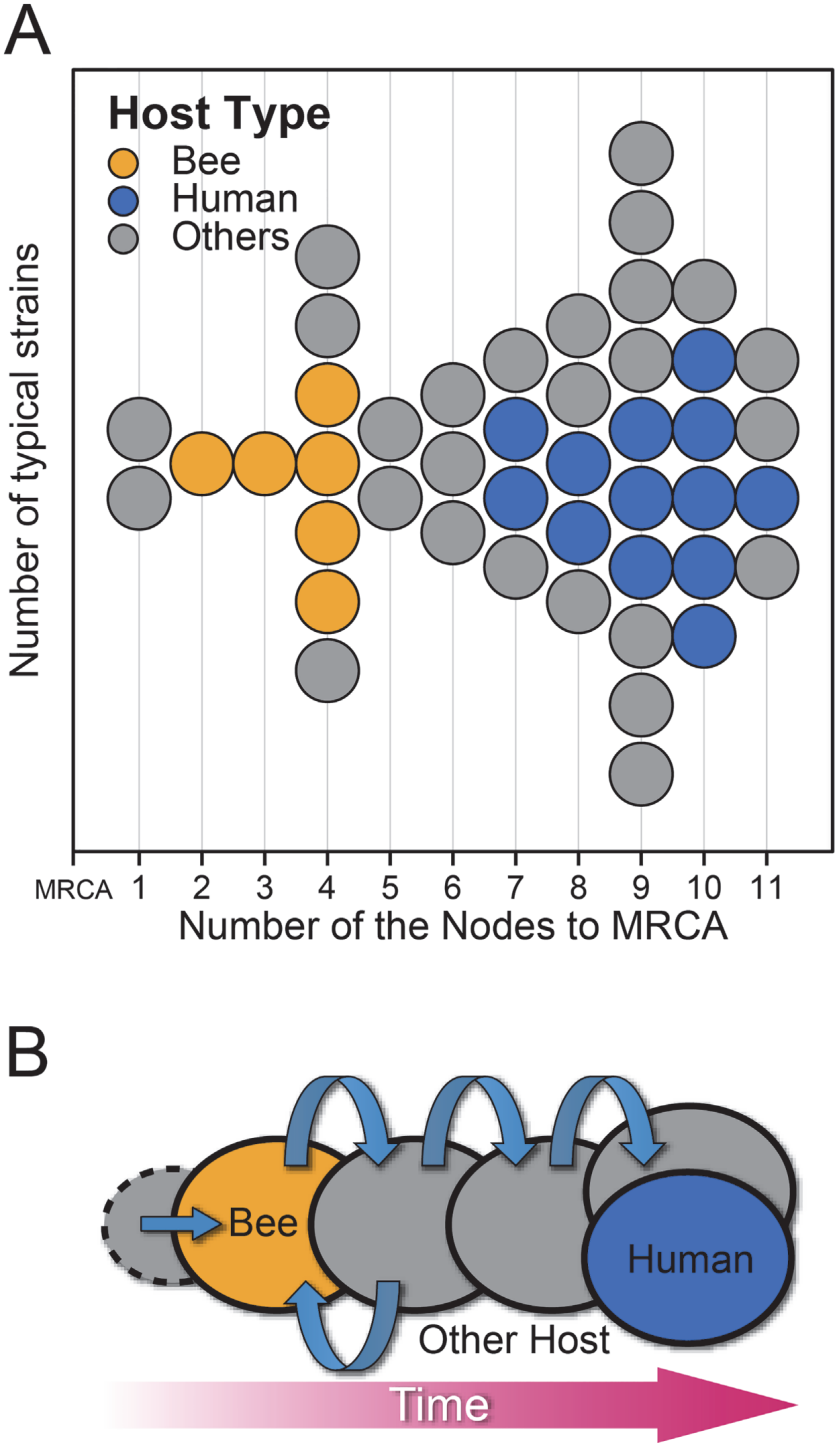
Unbalanced distribution of bee and human isolates in the genealogy. A: Number of nodes from each strain to the MRCA. The numbers were counted based on the maximum likelihood tree of the 45 type strains (Fig. 3). Each circle represents one strain. The color of the circle indicates the host type, with yellow for bee isolates, blue for human isolates and grey for isolates from the other sources. B: The hypothetical evolutionary scenario of the genus *Bifidobacterium*. The ellipses represent different hosts, and the arrows represent possible trans-hosts spreading.

Interestingly, when we related the hosts of bifidobacteria to the lineages, we found that the distribution of bee and human isolates was clearly unbalanced in the genealogy. Four of the five species in the most ancient lineage, including *B*. *actinocoloniiforme*, *B*. *asteroides*, *B*. *coryneforme* and *B*. *indicum*, were isolated from the bee gastrointestinal tract (Fig. 3). Two more bee isolates, *B*. *bombi* and *B*. *bohemicum*, were also located nearby the base of the MLtree. In contrast, all 15 human isolates were distributed among the younger lineages: 14 formed 2 clusters, with *B*. *gallicum* clustering with the *B*. *pseudolongum* group. We further measured the unbalanced distribution between bee and human isolates by comparing the number of nodes from each strain to the MRCA of the entire genus (Fig. 4A). The number of nodes to the MRCA for each species can be used to represent the number of speciation events over time, whereby a lower node number indicates fewer branching events since its ancestor and hence belonging to a more ancient lineage. Fig. 4A indicates that all six strains isolated from bees were much closer to the MRCA than the human isolates. Although only a limited number of strains isolated from bee and human were analyzed in this research, statistical tests on the number of nodes to the MRCA supported that the human isolates are much younger than the bee isolates (Wilcoxon Two-Sample test, P = 0.003). Therefore, bifidobacteria adapted to bees during the early history of evolution and then spread and colonized humans.

Based on a comparative genomic analysis, Bottacini et al. [37] have speculated that insects are the original host of bifidobacteria. However, we observed that two species, *B*. *psychraerophilum* and *B*. *minimum*, isolated from swine and sewage, respectively, are immediately descended from the MRCA and hence represent the most ancient lineage of bifidobacteria (Figs. 3 and 4A). Therefore, our results indicated that although bee isolates dominated the ancient lineage, bees or other insects are not necessarily the original hosts of bifidobacteria. Considering that the type strains isolated from other hosts were scattered between the bee and human isolates, one hypothetical evolutionary scenario is that bifidobacteria experienced numerous host jumping, first spreading from the original host (perhaps swine) to bees, then to non-primate animals and poultry, and finally to monkeys and humans (Fig. 4B). Additional genome sequences from bifidobacterial strains distributed in a wider range of hosts are needed to verify this hypothesis.

### Host-specific gene pools

The direct living environment of an organism may specifically shape its genome composition during evolution and ecological adaptation. Thus, by determining host-specific gene pools, we can identify genes that are involved in adapting to a certain niche or host. For further analysis, we only compared the type strains isolated from the gastrointestinal tracts of humans, bees and swine. The isolates from other sources were excluded from the analysis because of either limited sample size (n ≤ 4 for strains isolated from poultry, monkey, rabbit, bovine and rat) or uncertainty of the original source, e.g., strains isolated from sewage possibly originated from fecal contamination. We also excluded one strain each from human blood and the buccal cavity, as their distinct environments are rather different compared with the GI tract, an aspect that may uniquely shape the genome content and hence lead to possible bias in the analysis.

**Fig 5:**
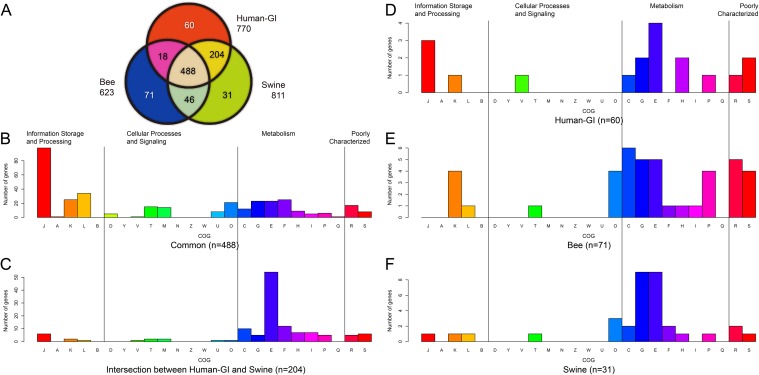
Functional categories of the host-specific gene families. Type strains isolated from bees, human-GI and swine were included in the analysis. A: Venn plot indicating the number of shared and unique core gene families of bifidobacterial strains from different hosts. B. The COG functional category of 488 shared core gene families by the three groups. C. The functional category of 204 core gene families shared by human-GI and swine. D-F: The functional category of host-specific core gene families from human-GI (n = 60), bee (n = 71) and swine (n = 31) separately.

To acquire the genes essential for each of the three bifidobacterial groups defined based on their host, we first constructed core-gene families for each group separately. The numbers of core-gene families were 623 (isolates from bees), 770 (humans) and 811 (swine), respectively (Fig. 5A). We then compared these core-gene families to profile their commonly shared and unique gene family sets. A total of 488 core-gene families were shared by all three groups, which was larger than the number of the core-gene families of the entire genus (n = 402) but composed of similar functional categories (Figs. 2 and 5B). Notably, the isolates from bees had the largest number (n = 71) of unique core-gene families, which included a set of genes encoding cytochrome d oxidases (*cydA*, *cydB*, *cydC* and *cydD*) and NADH dehydrogenase (*ndh1*) (S5 Table). In *B*. *asterodies*, these genes are putatively involved in respiratory metabolism, which predicts the ability of this species to survive in an oxygenated host [37]. As the insect gut is an oxygen-rich niche, in contrast to that of mammals/birds [38], the presence of these aerobic metabolism-associated genes appears to be necessary for the adaptation of insect-originating bifidobacteria to their environment. The results also suggested that the ancestor of *Bifidobacterium* might have survived in an open oxygen-rich environment because of the dominance of bee isolates in the oldest lineage, which preserved the ancestral characteristics.

A large number (n = 204) of core-gene families overlapped between the species isolated from the GI tract of humans and swine (Fig. 5A). This is expected because of the high similarity of the gut niche of humans and swine compared to that of insects. We also investigated the function of the specific core-gene families of the bifidobacteria group based on their host according to their COG category assignment (Fig. 5D, 5E, 5F and S5 Table). Although potential bias may have resulted from the limited number of genes annotated with COG categories for each group, we nonetheless observed a clear pattern of the enrichment of core genes relating to the category of metabolism (Fig. 5C, 5D, 5E and 5F). Genetic adaptation to specific carbohydrate and amino acid metabolism has been reported in *B*. *longum* [39], *B*. *animalis* [40], and *B*. *dentium* [26]. Therefore, the unique metabolism-associated gene profiles for each group may reflect the specific nutrient environment in the gastrointestinal tract of the different hosts.

### Species taxonomy based on whole-genome sequences

The combination of ANI values, AAI values and core-genome phylogeny can provide comprehensive taxonomy guidance for bacteria with respect to whole-genome sequences [41]. According to the genome distance revealed by ANI, AAI and the phylogenetic relationship shown in the core-gene tree, we propose three revisions of the current species designation system for bifidobacteria. First, *B*. *indicum* and *B*. *coryneforme* originally belonged to the same phylogroup, namely *B*. *asteroides*, and previously were regarded as two distinct species [24]. Here, we show that the pair-wise ANI value between them is 98.27% (AAI, 98.39%) (S3, S4 Figs. and S6 Table), which is much higher than the cut-off value of 95% distinguishing different species [41]. Thus, it would be more appropriate if *B*. *indicum* and *B*. *coryneforme* are combined as a single species based on their genetic distance. A similar argument for species reclassification based on ANI values applies to *B*. *saeculare* and *B*. *gallinarum* from phylogroup *B*. *pullorum* as well as *B*. *stercoris* and *B*. *adolescentis* from phylogroup *B*. *adolescentis*. In previous research based on the sequences of conserved genes [42, 43], these two pairs of species were each considered to belong to the same species. As they showed ANI values of 96.79% (AAI, 95.29%) and 97.69% (AAI, 96.78%), respectively, the sequence identity at the whole-genome level also supports previous proposals to combine them into the same species.

## Conclusions

In the present study, 45 type strains covering 93.75% of the genus *Bifidobacterium* were sequenced and analyzed. According to our comparative genomic analysis, the results depict a snapshot of the genetic diversity and evolutionary history of this genus, which will pave the way for future studies on their taxonomy and functional genomics.

Our data show that although the ancestor of bifidobacteria most likely colonized bees at the very beginning stage of evolution, bees or other insects might not be the original host of this genus. The results also provide a hypothetical evolutionary scenario of bifidobacteria in which multiple host jumping events occurred during the spread from bees to humans. Furthermore, we observed that the species isolated from different types of host possess unique gene sets, which may be associated with the host-specific niche, such as the association between the oxygenated gut environment of bees and cytochrome d oxidases. However, as only type strains were sequenced in this study, the sample size for each host type was still limited, which may have hindered further interpretation of the adaptation of bifidobacteria to specific niches. More isolates from a wider range of host types and ecological niches are thus needed to provide in-depth insight into the evolutionary scenario of this human probiotic genus.

## Supporting Information

S1 Fig Phylogenetic position of *Bifidobacterium* in the tree of bacteria. The tree was built based on 31 marker genes shared by 471 strains (including 45 *Bifidobacterium* species, shown in red) by the maximum likelihood method using PHYML with the WAG model.
(EPS)

S2 Fig Phylogeny of the 45 *Bifidobacterium* genomes and 2 representative genomes of *Gardnerella* and *Brachybacterium* based on 185 core genes by the maximum likelihood method using PHYML with the WAG model. (EPS)

S3 Fig Pairwise ANI values across 45 *Bifidobacterium* genomes. The order of these strains is sorted according to their positions in the phylogenetic tree based on the maximum likelihood tree of the 45 type strains (Fig. 3). The colors in heat map represent pairwise ANI values, with black for the ones larger than 95% and gradient colors from red (low identity) to blue (high identity) for the others.
(EPS)

S4 Fig Pairwise AAI values across 45 *Bifidobacterium* genomes. The order of these strains is sorted according to their positions in the phylogenetic tree based on the maximum likelihood tree of the 45 type strains (Fig. 3). The colors in heat map represent pairwise AAI values, with black for the ones larger than 95% and gradient colors from red (low identity) to blue (high identity) for the others.
(EPS)

S1 Table Background of the strains sequenced in the current research. (DOCX)

S2 Table General genomic features of the type strains. (XLSX)

S3 Table General information of the core genes shared by 45 *Bifidobacterium* type strains. (XLSX)

S4 Table Information of 426 representative genomes used in building the tree for bacteria. (XLSX)

S5 Table Functional annotation of host-specific gene families. (XLSX)

S6 Table Pairwise ANI and AAI values across 45 *Bifidobacterium* genomes. (XLSX)
