# Supplementary material for: Comparative Genomic Analysis of 45 Type Strains of the Genus Bifidobacterium: A Snapshot of Its Genetic Diversity and Evolution
Source: PLoS One. 2015 Feb 6;10(2):e0117912. doi: 10.1371/journal.pone.0117912 (PMC4319941; doi:10.1371/journal.pone.0117912)
Supplement: S1 Table — (DOCX) [file pone.0117912.s005.docx]

**Table S1 Background of the strains sequenced in the current research**

| **Species** | **Identification number*** | **Source** | **Reference** |
| --- | --- | --- | --- |
| *B. actinocoloniiforme* | DSM 22766 | Bumblebee digestive tract | [1] |
| *B. angulatum* | DSM 20098 | Human faeces | [2] |
| *B. asteroides* | DSM 20089 | Intestine of honeybee | [3] |
| *B. biavatii* | DSM 23969 | Faeces of red-handed tamarind | [4] |
| *B. bifidum* | DSM 20456 | Infant feces | [3] |
| *B. bohemicum* | DSM 22767 | Bumblebee digestive tract | [1] |
| *B. bombi* | DSM 19703 | Bumblebee digestive tract | [3] |
| *B. boum* | DSM 20432 | Rumen of cattle | [3] |
| *B. breve* | DSM 20213 | Intestine of infant | [3] |
| *B. callitrichos* | DSM 23973 | Marmoset faeces | [4] |
| *B. catenulatum* | DSM 16992 | Intestine of adult | [2] |
| *B. choerinum* | DSM 20434 | Porcine feces | [3] |
| *B. coryneforme* | DSM 20216 | Intestine of honeybee | [3] |
| *B. cuniculi* | DSM 20435 | Rabbit feces | [3] |
| *B. dentium* | DSM 20436 | Human dental caries | [2] |
| *B. gallicum* | DSM 20093 | Human feces | [3] |
| *B. gallinarum* | DSM 20670 | Chicken cecum | [3] |
| *B. indicum* | DSM 20214 | Intestine of honeybee | [3] |
| *B. kashiwanohense* | DSM 21854 | Faeces of infant | [3] |
| *B. longum* subsp. *infantis* | DSM 20088 | Intestine of infant | [3] |
| *B. longum* subsp. *suis* | DSM 20211 | Porcine feces | [3] |
| *B. magnum* | DSM 20222 | Rabbit feces | [3] |
| *B. merycicum* | DSM 6492 | Bovine rumen | [3] |
| *B. minimum* | DSM 20102 | Sewage | [3] |
| *B. mongoliense* | DSM 21395 | Airag, traditional beverage made of fermented mare's milk | [5] |
| *B. pseudocatenulatum* | DSM 20438 | Infant feces | [3] |
| *B. pseudolongum* subsp. *globosum* | DSM 20092 | Bovine rumen | [3] |
| *B. pseudolongum* subsp. *pseudolongum* | DSM 20099 | Porcine feces | [3] |
| *B. psychraerophilum* | DSM 22366 | Porcine feces | [3] |
| *B. pullorum* | DSM 20433 | Chicken feces | [3] |
| *B. reuteri* | DSM 23975 | Marmoset faeces | [4] |
| *B. ruminantium* | DSM 6489 | Bovine rumen | [3] |
| *B. saeculare* | DSM 6531 | Rabbit feces | [3] |
| *B. saguini* | DSM 23967 | Faeces of red-handed tamarind | [4] |
| *B. scardovii* | DSM 13734 | Human blood | [3] |
| *B. stercoris* | JCM 15918 | Human faeces | [6] |
| *B. subtile* | DSM 20096 | Sewage | [3] |
| *B. thermacidophilum* subsp. *porcinum* | DSM 17755 | Porcine feces | [3] |
| *B. thermacidophilum* subsp. *thermacidophilum* | DSM 15837 | Sewage | [3] |
| *B. thermophilum* | DSM 20210 | Porcine feces | [3] |
| *B. tsurumiense* | DSM 17777 | Dental plaque of hamster | [7] |
| *B. animalis* subsp. *animalis* | ATCC 25527 | Rat faeces | [3] |
| *B. animalis* subsp. *lactis* | ATCC 27653 | Yogurt | [3] |
| *B. adolescentis* | ATCC 15703 | Intestine of adult | [3] |
| *B. longum* subsp. *longum* | JCM 1217 | Intestine of adult | [3] |

*: DSM, the strains were acquired from German Collection of Microorganisms and Cell Cultures; JCM, the strains were acquired from Japan Collection of Microorganisms; ATCC, the strains were acquired from American Type Culture Collection.

References

1. Killer J, Kopecny J, Mrazek J, Koppova I, Havlik J, et al. (2010) *Bifidobacterium actinocoloniiforme* sp. nov. and *Bifidobacterium bohemicum* sp. nov., from the bumblebee digestive tract. Int J Syst Evol Microbiol 61: 1315-1321.
2. Scardovi V, Crociani F (1974) *Bifidobacterium catenulatum*, *Bifidobacterium dentium*, and *Bifidobacterium angulatum*: three new species and their deoxyribonucleic acid homology relationships. Int J Syst Bacteriol 24: 6-20.
3. Lee JH, Sullivan DJO (2010) Genomics insights into *Bifidobacteria*. Microbiol Mol Biol Rev 74: 378-416.
4. Endo A, Futagawa-Endo Y, Schumann P, Pukall R, Dicks LM (2012) *Bifidobacterium reuteri* sp. nov., *Bifidobacterium callitrichos* sp. nov., *Bifidobacterium saguini* sp. nov., *Bifidobacterium stellenboschense* sp. nov. and *Bifidobacterium biavatii* sp. nov. isolated from faeces of common marmoset (*Callithrix jacchus*) and red-handed tamarin (*Saguinus midas*). Syst Appl Microbiol 35: 92-97.
5. Watanabe K, Makino H, Sasamoto M, Kudo Y, Fujimoto J, et al. (2009) *Bifidobacterium mongoliense* sp. nov., from airag, a traditional fermented mare's milk product from Mongolia. Int J Syst Evol Microbiol 59: 1535-1540.
6. Kim MS, Roh SW, Bae JW (2010) *Bifidobacterium stercoris* sp. nov., isolated from human faeces. Int J Syst Evol Microbiol 60: 2823-2827.
7. Okamoto M, Benno Y, Leung KP, Maeda N (2008) *Bifidobacterium tsurumiense* sp. nov., from hamster dental plaque. Int J Syst Evol Microbiol 58: 144-148.
